# The Influence of Diet, Water Intake, Exercise, Education Level, and Income on the Quality of Sleep in the Saudi Population: A Cross-Sectional Study

**DOI:** 10.7759/cureus.24074

**Published:** 2022-04-12

**Authors:** Omar Tarabzoni, Ahmad M Samman, Ahmed Hilabi, Abdullah Alrasheed, Mohammed A Alkhamis, Mohammed S Alnumani, Feras S Al harbi, Abdulrahman Alraddadi, Awad M Almuklass

**Affiliations:** 1 College of Medicine, King Saud Bin Abdulaziz University for Health Sciences, Riyadh, SAU; 2 Basic Medical Sciences, Anatomy, King Saud Bin Abdulaziz University for Health Sciences, Riyadh, SAU; 3 Research and Development, King Abdullah International Medical Research Center, Riyadh, SAU

**Keywords:** saudi population, income, educational level, water intake, social status, diet, insomnia, the quality of sleep

## Abstract

Background: Sleep quality is known to affect and be affected by multiple factors in one’s life through physiological and psychological manners. The study aimed to assess the influence of diet, water intake, exercise, level of education, and income on the quality of sleep of the Saudi population.

Materials and methods: The study was a cross-sectional design that used a validated standardized fact-based questionnaire developed based on the Insomnia Severity Index. The data collection continued for three months, during which subjects were approached and interviewed in many locations, ensuring the Saudi population's best representation.

Results: The study included 496 subjects, of whom 74% were men. Most of the participants (51.4%) were aged between 18 and 25 years old and were unmarried (64.2%). The education level of participants was between high school and bachelor's level (45-43%), respectively. Around 53% believed that they were following an unhealthy diet, although 42% of the total had a normal BMI. About 44% of participants consumed about 1-2 L of water daily, and 42% never exercised. The majority (39%) earned an income of >5000 Saudi Riyals. Forty-two percent of the participants reported subthreshold insomnia. The data analysis showed that social status and diet were significantly related to sleeping quality (P = 0.051, P = 0.027). Additionally, the level of education was found to be an important confounding factor.

Conclusion: Diet and social status were significantly related to insomnia, and educational level served as a significant confounding factor. Neither water intake, exercise, BMI category, age, nor gender were associated with insomnia.

## Introduction

Insomnia is clinically defined by the Diagnostic and Statistical Manual of Mental Disorders (DSM-5) as difficulty initiating or maintaining sleep, or early-morning awakening that leads to dissatisfaction with sleep quantity or quality. The definition excludes any sleep disturbances due to mental disorders and medical conditions [1]. Several studies discussed the global prevalence of insomnia. The percentages ranged from 10% up to 60% [2]. Sleep is an important factor that has a profound effect on multiple aspects of health, both physical and psychological, growth, education, and functionality of the individual. Good sleep behavior can improve the overall health and reduces the risk of chronic disease development, boosts mood and energy, enhances both brain and immune function, and ensure healthy growth and development of the young [3]. Sleep deprivation is linked to multiple health problems, including high blood pressure, obesity, and type 2 diabetes. Moreover, lack of sleep is associated with memory issues, defective thinking processes, weakened immunity, reduced libido, and an increased frequency of accidents [4]. Studies concerned with sleep, growth, and education also found that insomnia is associated with decreased attention, impaired memory, slowed processing, decreased creativity, and excessive daytime sleepiness, which reduces overall school performance [5].

Age and its related processes have been essential demographic factors in many studies, as they can change multiple aspects, such as treatment and prognosis [6]. Therefore, it is appropriate to highlight the association between aging and insomnia. In addition to age, gender is another vital factor that needs to be investigated when it comes to insomnia since the female body goes through multiple physiological changes, making it prone to variation in the sleeping pattern [7]. The marital status of a person has a complex relationship with a person's functionality as humans differ in their personalities, and this relationship has been studied and found to cause suboptimal function [8]. Many studies rendered the educational level insignificant regarding insomnia [9], while others found a weak link to health-related issues [10].

Diet is considered a critical factor that influences one's life through multiple pathways. For example, diet can affect and be affected by people's mental, physiological, and social status. A diet can be classified as healthy or unhealthy based on the quality, quantity, and caloric intake based on the supply of macronutrients (carbohydrates, proteins, and fats) contained in the food [11].

Water intake is one of the essential indicators of a person's diet and health, as around 60% of our bodies are composed of water and can only function if there is enough. Besides hydration, there are various disease pathophysiology processes related to the amount of water intake. Physical exercise is vital to affecting calorie intake, and it is considered one of the effective non-pharmacological therapy for insomnia [12]. It is readily available and cost-free. For instance, a randomized controlled trial (RCT) confirmed that physical exercise positively affects sleep quality, sleep onset latency, total sleep time, sleep efficiency, and insomnia severity [13]. In recent years, many studies have discussed the factors related to insomnia. However, none were local, which led us to the aim of this research, which extensively investigates the relationship between insomnia, age, gender, diet, water intake, exercise, social status, and level of education in the Saudi population.

## Materials and methods

Setting and participants

The study targeted the Saudi population of Riyadh City, Kingdom of Saudi Arabia. After obtaining the approval of the Institutional Review Board at the Ministry of National Guard-Health Affairs SP20/003/R, data collection took place in various public areas to target the general population with no preference (public areas such as malls, hospitals, parks, scientific gatherings, universities, and mosques) during the period January 2020 to March 2020. The study participants included males and females from various age groups, which were classified into: 18-25 years old, 26-40 years old, 41-50 years old, and >50. The participants were divided into married and unmarried, which included single, divorced, and widowed. Regarding the educational level, the classification of the subjects depends on the highest level achieved (higher education, bachelor, high school, or less than high school, which includes elementary, intermediate, and no education). The diet plan followed by the subjects was further divided into healthy and unhealthy categories according to the participant’s perspective. The body mass index (BMI) groups were (under-weight, normal, over-weight, obese). The amount of water consumed was classified into <1 L per day, 1-2 L per day, 2-3 L per day, and >3 L per day. The frequency of physical exercise conducted by the participants was: never exercised, once a week, twice a week, >2 times per week. The monthly income was categorized into: <5000 Saudi Riyal, 5000-10.000 SR, 10.000-20.000 SR, >20.000 SR, and preferred not to answer). The sampling technique used was convenience sampling using Raosoft software (Raosoft, Inc., Seattle, WA).

Questionnaire

After obtaining their consent, investigators educated the participants on the research parameters, followed by the participants filling out a questionnaire that assessed their quality of life based on the World Health Organization Quality of Life (WHOQOL) BREF questionnaire [14]. The questionnaire included English and Arabic sections based on five domains (general health, social and financial status, physical activities, general diet, and sleep health) utilizing fact-based questions only. The questionnaire included a brief medical history regarding chronic diseases and general physical examinations, including height and body weight measurements. The questionnaire inquired about BMI by measuring weight (kg) and then dividing it by the square of height (m).

Statistical analysis

The sample size was calculated using Raosoft software. The margin of error was set at 5%, with a confidence level of 95%. The questionnaires were coded, and the data collected were entered into Excel 2020 files. All data were analyzed using SPSS v27 software. Categorical data were presented as frequencies and percentages. The Chi-square test, independent t-test, one-way ANOVA, and Binary (Binomial) Logistic Regression were used. Any P-value ≤0.05 was considered significant. Missing data were handled by eliminating the missing values through the innate programming of the statistical software during analysis.

## Results

The study included 496 subjects, of whom the majority (N=367, 74%) were men. Most of the participants were young people aged between 18 and 25 years old (N=257, 51%). The remaining subjects were categorized into the following age groups: 26 to 40 years (N=175, 35%), 41 to 50 years (N=38, 8%), and over 50 years (N=31%). The subjects were mostly unmarried (N=323, 64%). The level of education in most subjects was between high school and bachelor's (45% and 43%, respectively). Only 34 of the participants (7%) had a higher education, and the minority had an educational level of less than high school (N=26, 5%). More than half of the participants (N=263, 53%) believed they followed an unhealthy diet. However, after measuring the BMI, the majority were in the "normal" range (N=201, 42%), followed by the overweight category (N=147, 31%; BMI = 25.0-29.9 kg/m^2^), obese category (N=103, 21%); BMI ≥ 30.0 kg/m^2^), and finally the underweight category (N=30, 6%; BMI < 18.5 kg/m2).

Concerning water intake, most of the participants (N= 220, 44%) consumed about 1-2 L of water daily, followed by less than 1 L/daily and 2-3 L/daily (N=122, 24% and N=117, 23%, respectively). Furthermore, evaluating the fitness level of the participants showed that most of the subjects (N=210, 42%) "never exercised," but around 27% (N=132) exercised more than twice/week, followed by once and twice/week (N=79, 16% and N=75, 15%, respectively). 

With respect to the income level of the participants, the survey result showed that most of the participants received an income of less than five thousand Saudi Riyal (N=195, 39%). Around 16% of participants (N= 81) received 10-20k, followed by those who received 5-10k (N=69, 14%) and more than 20k (N=33, 7%). However, a quarter of the participants (N=126, 25%) preferred not to answer (Table 1).

**Table 1:** Demonstrate demographics including gender, age, marital status, educational level, BMI and income. Also, health determinant like exercise, diet, and water intake of the participants and their percentages. *Unmarried category included singles, divorced, and widowed. **Diet was answered upon participant point of view.

Variables		N (%)
Gender	Male	367 (74)
	Female	129 (26)
Age category	18-25	257 (51)
	26-40	175 (35)
	41-50	38 (8)
	51+	30 (6)
Social status	Unmarried*	323 (64)
	Married	180 (36)
Level of education	Less than high school	26 (5)
	High school	227 (45)
	Bachelor	217 (43)
	Higher education	34 (7)
BMI	Underweight	30 (6)
	Normal	201 (42)
	Overweight	147 (31)
	Obese‎	103 (21)
Income	Prefer not to answer	126 (25)
	Less than 5k	195 (39)
	5-10k	69 (14)
	10-20k	81 (16)
	More than 20k	33 (7)
Exercise	Never	210 (42)
	Once a week	79 (16)
	Twice a week	75 (15)
	More than twice per week	132 (27)
Diet**	Unhealthy	263 (53)
	Healthy	234 (47)
Water-intake	Less than 1 liter per day	122 (24)
	1-2 liters per day	220 (44)
	2-3 liters per day	117 (23)
	More than 3 liters per day	41 (8)

Among the variables measured, the data analysis showed that diet, social status, and educational level had a significant correlation to sleeping quality (Table 2). There were more subjects in the unhealthy diet group (19%) who reported clinical insomnia than those in the healthy diet group (10%) (P=0.002, OR=1.85). Regarding social status, the unmarried subjects (19%) reported more clinical insomnia than the married subjects: 19% compared to 10% (P=0.004, OR=1.87). The level of education showed variable effects on the quality of sleep of the participants. There were more bachelor holders (18%) who reported having clinical insomnia than those with higher education (3%) (P=0.011). However, there was no difference between the other groups according to the education level (P>0.050) (Table 3).

**Table 2:** The correlation between all variables and different stages of insomnia based on ISI questionnaire. *NCSI: No clinically significant insomnia, STI: sub-threshold insomnia, CI: clinical insomnia.

Variables		NCSI	STI	CS	P-value
		N (%)	N (%)	N (%)	
Gender	Male	142 (40)	157 (45)	54 (15)	0.856
	Female	53 (42)	52 (42)	20 (16)	
Age category	18-25	87 (35)	116 (47)	45 (18)	0.114
	26-40	75 (44)	69 (41)	25 (15)	
	41-50	19 (54)	13 (37)	3 (9)	
	51+	16 (53)	12 (40)	2 (7)	
Social status	Unmarried	114 (36)	142 (45)	58 (19)	0.004*
	Married	85 (50)	68 (40)	17 (10)	
Level of education	Less than high school	17 (65)	7 (27)	2 (8)	0.011*
	High school	77 (36)	106 (49)	34 (16)	
	Bachelor	86 (41)	85 (41)	38 (18)	
	Higher education	19 (58)	13 (39)	1 (3)	
BMI	Underweight	11 (38)	12 (41)	6 (21)	0.811
	Normal	83 (42)	86 (44)	28 (14)	
	Overweight	57 (40)	64 (45)	20 (14)	
	Obese	44 (44)	37 (37)	19 (19)	
Income	Prefer not to answer	49 (41)	47 (40)	23 (19)	0.386
	< 5k	73 (39)	87 (47)	27 (14)	
	5-10k	26 (38)	28 (41)	14 (21)	
	10-20k	35 (43)	39 (48)	7 (9)	
	> 20k	16 (53)	10 (33)	4 (13)	
Exercise	Never	73 (36)	91 (45)	39 (19)	0.105
	Once a week	29 (38)	36 (47)	12 (16)	
	Twice a week	32 (45)	26 (37)	13 (18)	
	More than twice per week	62 (48)	57 (44)	11 (9)	
Diet	Unhealthy	88 (35)	117 (46)	49 (19)	0.002*
	Healthy	109 (48)	93 (41)	23 (10)	
Water-intake	< 1 L/day	40 (35)	54 (47)	22 (19)	0.57
	1-2 L/day	97 (45)	88 (41)	30 (14)	
	2-3 L\day	43 (38)	52 (46)	17 (15)	
	< 3 L\day	19 (46)	16 (39)	6 (15)	

**Table 3:** Demonstrate the odds ratio (OR) between the variable and their references. *References value is 1.

Variables		P-value	OR	95% C.I.	
				Lower	Upper
Social-status	Unmarried	0.051	1.87	1	3.52
	Married*	REF	1		
Level of education	Educational-level (1)	0.554	2.12	0.18	25.36
	Educational-level (2)	0.256	3.31	0.42	25.98
	Educational-level (3)	0.11	5.26	0.69	40.27
		REF	1		
Diet	Unhealthy	0.027	1.85	1.07	3.19
	Healthy*	REF	1		

## Discussion

In this study, diet, water intake, physical exercise, income, BMI, gender, age, social status, and educational level were investigated to study the correlation with insomnia. The results of the study suggest that diet (P=0.002), social status (P=0.004), and level of education (P=0.011) can significantly affect sleep quality and insomnia.

Insomnia and diet

Compared to the healthy diet group, the ones who adopted an unhealthy diet had a higher possibility of developing clinical insomnia and subthreshold insomnia (Table 2). The results of this study are consistent with previous work. A previous prospective cohort study of post-menopausal women who participated in the Women's Health Initiative found that a high glycemic diet could be a risk factor for developing clinical insomnia [15]. In contrast, a cross-sectional study that investigated the relationship between the presence of insomnia, physical activity, and diet quality in Spanish adults could not find any significant relationship between diet quality and insomnia [16]. This result could be attributed to the small sample size involved in the study.

Water intake and insomnia

The ambiguous relationship between water intake and insomnia is still not fully understood. It could be explained by the dynamic complexity of the water regulatory network and inter-individual differences in the body's normal functioning, including sleeping patterns [17]. However, the current study investigated the relationship between water intake and insomnia, showing no statistically significant (p=0.57) association between the two. Thus, further studies are recommended to explore the complexity of the association between water intake and sleep quality.

Physical activity and insomnia

Good physical activity is known to improve overall health in multiple ways. One of which is the prevention and management of non-communicable diseases such as heart disease, stroke, and diabetes. It also improves muscular and cardio-respiratory fitness [18]. The current study shows no association between insomnia and physical activity. Moreover, both physical exercise and light therapy [19] had less convincing evidence and were only weakly recommended by the European Sleep Research Society [20]. On the other hand, a cross-sectional study establishes a bivariant correlation where insomnia could develop if a subject had moderate-intense physical exercise for longer than 120 minutes or less than 20 minutes per day [21]. This study is consistent with previous studies in which the association was not significant either way. Further research is needed to explore the relationships in the Saudi population.

Body mass index and insomnia

A body mass index is used as an estimation of the overall physical health and status of an individual. The current study is consistent with previous studies; BMI has not been shown to be significantly correlated with insomnia. For example, a study stated that BMI did not explain sleep difficulties and insomnia symptoms [22]. Nevertheless, an unexpected significant correlation between lower BMI and severe chronic insomnia among 233 German adults was found [23].

Gender and insomnia

Both sex and age play a significant role in insomnia [24]. A previous study showed a correlation between the female gender and insomnia that can be explained by a complicated multifactorial interplay between biological, psychological, and social factors [24]. The biological aspect may be explained by the hormonal changes associated with the beginning of menstruation, as witnessed by some studies which found that the risk of insomnia in females starts to increase with the beginning of their menses and the hormonal changes in both estrogen and progesterone that occur with it [25]. Additionally, there are predispositions to certain physical and mental issues that affect women as an overall prevalence. The sleep problem does not end with the cessation of the periods, but disturbances in hormone levels due to any reason, such as changes in circadian rhythm, as well as the accompanying symptoms like hot flashes and night sweats, can also lead to insomnia. Additionally, the risk of insomnia in females is further increased by stressors such as the disparities in social and cultural aspects [25]. However, the current study did not detect any relationship between the gender of participants and insomnia, which could be attributed to the male to female ratio of 3:1 in our study sample.

Age and insomnia

Insomnia in older adults is often considered a normal aging phenomenon, yet the increased prevalence of chronic conditions in later life may explain most of the insomnia symptoms in the older population [24]. Only 1% to 7% of insomnia in later life occurs independently of chronic conditions [24]. Up to 50% of older adults report insomnia symptoms; this casts a shadow of doubt over the notion of insomnia being a part of the normal aging process [24]. The current study shows no significant association between age and insomnia. Hence, consistent with others, the current study supports that age does not cause insomnia, but the presence of other comorbidities may lead to insomnia in older adults [26].

Social status and insomnia

Social status represents multiple aspects that act as pillars in an individual's life. One of the aspects that highly affects the prestige of a person is their marital status, which in many cultures can be seen as the definition of manhood, the establishment of a family, and taking part in the overall social hierarchy. Marriage holds a great position because of its role in building mental and physical stability through partnership and the production of the future generation. Social status was significantly related to insomnia in this study. Those who were not married were more likely to suffer from insomnia. A cross-sectional study conducted on 1490 incarcerated adults manifested that around 51% of subjects with insomnia were unmarried [27]. On the contrary, a cross-sectional study among 3045 participants found that around 727 had insomnia, and approximately 77% were married [28]. Being married can offer social support, which can have an impact on someone’s mental and physical health that can manifest as insomnia if absent. However, cultural differences regarding marriage and its responsibilities can display different findings in other populations.

Educational level and insomnia

The educational level can determine many factors shaping one’s life and choices. In our study, educational level was only found to act as a confounding factor and not as an independent, statistically significant factor. In another study, lower levels of education may contribute to insomnia in some cases [24].

Monthly income and insomnia

Different monthly incomes in the population of the study were not related by any means to insomnia. On the other hand, a study that was done by Lewis found a positive correlation between monthly income and insomnia, whether in the family or the individual [29]. However, a cross-sectional study conducted in Pakistan concluded that increased income was associated with the development of insomnia [30].

Limitations and recommendations

The study had a couple of limitations. The study took place just before the pandemic of COVID-19, and with the COVID-19 social measures and restrictions, there were challenges faced that impacted data collection. It is recommended that more studies be conducted to measure the effect of age and gender on the development of insomnia in the Saudi population since no correlation was detected in the current study. The level of education was found to be a confounding factor, and more research should focus on the logic behind the development of insomnia in undergraduate students. Marriage has a complex nature depending on the culture and society, which is recommended to be studied in detail.

## Conclusions

Finally, the study looked at a variety of personal and life factors that influence insomnia development in the general population. Diet, water consumption, physical activity, BMI, age, gender, degree of education, social status, and income were all studied. Only three characteristics were shown to have a significant relationship with the development of insomnia: diet, social status, and educational level. People who maintained a poor diet had a greater risk of acquiring clinical and subthreshold insomnia than those who followed a good diet. In this study, social status was found to be a major factor in sleeplessness. Being married can provide social support, which can have an influence on someone's mental and physical health and, if not provided, might aid in the development of insomnia. However, cultural variances in attitudes about marriage and its responsibilities can lead to different results in various groups, so it is advised that it be researched in depth. The degree of education was discovered to be a confounding factor, and additional research into the causes of insomnia among undergraduate students is needed.
